# Albumin‐Bilirubin Score as a Novel Prognostic Prediction Tool for Surgically Treated Head and Neck Squamous Cell Carcinoma

**DOI:** 10.1002/kjm2.70115

**Published:** 2025-09-25

**Authors:** Ming‐Hsien Tsai, Chao‐Hui Yang, Yu‐Tsai Lin, Hui‐Ching Chuang, Tai‐Lin Huang, Hui Lu, Wen‐Ling Tsai, Chih‐Yen Chien, Fu‐Min Fang

**Affiliations:** ^1^ Department of Otolaryngology Kaohsiung Chang Gung Memorial Hospital and Chang Gung University College of Medicine Kaohsiung Taiwan; ^2^ Graduate Institute of Clinical Medical Sciences College of Medicine, Chang Gung University Taoyuan Taiwan; ^3^ School of Medicine Chang Gung University College of Medicine Taoyuan Taiwan; ^4^ School of Medicine College of Medicine, National Sun Yat‐Sen University Kaohsiung Taiwan; ^5^ Department of Hematology and Oncology Kaohsiung Chang Gung Memorial Hospital and Chang Gung University College of Medicine Kaohsiung Taiwan; ^6^ Doctoral Program of Clinical and Experimental Medicine National Sun Yat‐Sen University Kaohsiung Taiwan; ^7^ Department of Cosmetics and Fashion Styling, Center for Environmental Toxin and Emerging‐Contaminant Research Cheng Shiu University Kaohsiung Taiwan; ^8^ Department of Radiation Oncology Kaohsiung Chang Gung Memorial Hospital, Chang Gung University College of Medicine Kaohsiung Taiwan

**Keywords:** ALBI, head and neck cancer, prognosis, serum albumin, serum bilirubin

## Abstract

We investigated the prognostic significance of the preoperative albumin‐bilirubin (ALBI) score in surgically treated head and neck squamous cell carcinoma (HNSCC). This retrospective study included 663 patients who underwent radical surgery between 2007 and 2017. The ALBI score, calculated using preoperative bilirubin and albumin levels, was assessed for its impact on overall survival (OS) through univariate and multivariate Cox regression analyses. Patients were randomly assigned to training and validation cohorts in a 3:1 ratio, and using a cutoff of −2.871, patients were stratified into low‐ and high‐ALBI groups, revealing significant prognostic differences in both cohorts. A high preoperative ALBI score was an independent predictor of worse OS in both cohorts, with an ALBI‐based nomogram being developed to predict OS with strong concordance indices (0.759 and 0.749 in the training and validation cohorts, respectively). Accordingly, the ALBI score is a simple and effective prognostic marker for improving risk stratification and survival prediction in HNSCC patients.

## 1 Introduction

Globally, head and neck squamous cell carcinoma (HNSCC) ranks as the sixth most prevalent cancer, while among Taiwanese males, it is the fourth most common malignancy [1, 2] HNSCC typically involves a multimodal approach, combining surgical excision, radiotherapy, and chemotherapy; although despite advancements in these modalities, the 5‐year survival rate for HNSCC patients remains at approximately 50%–60% [3]. Emerging evidence underscores the significance of host inflammatory and nutritional status in HNSCC development and treatment outcomes [4, 5, 6, 7, 8, 9]. There is growing recognition that inflammatory biomarkers reflect the complex interplay between the tumor microenvironment and the host immune response [10]. Several preoperative inflammatory biomarkers derived from peripheral blood cell counts, such as neutrophil‐to‐lymphocyte ratio, lymphocyte to monocyte ratio, platelet‐to‐lymphocyte ratio, or systemic inflammation response index, have shown associations with clinical outcomes in HNSCC [11, 12, 13, 14, 15, 16].

The albumin‐bilirubin (ALBI) score, introduced in 2015 as a simple and readily available tool for assessing liver function, has garnered attention [17]. Notably, the ALBI score has been identified as another surrogate marker for cancerogenic proinflammatory and immunosuppressive states in hepatocellular carcinoma (HCC) patients [18]; moreover, the ALBI score was capable of predicting the outcomes of several types of malignancy, including HCC [19], pancreatic cancer [20], colorectal cancer [21], gastric cancer [22], esophageal cancer [23], and non‐small cell lung cancer [24].

Nevertheless, data on the association between the ALBI score and survival outcomes in patients with HNSCC are still scarce. Accordingly, we conducted this study to investigate the prognostic value of the preoperative ALBI score in HNSCC patients and to establish a predictive nomogram for validation within our patient cohort.

## 2 Materials and Methods

### 2.1 Study Population

This retrospective study included 663 patients with histologically confirmed first primary HNSCC who underwent radical surgery at Kaohsiung Chang Gung Memorial Hospital, Taiwan, between January 2007 and February 2017. All patients had comprehensive clinical and pathological data available for analysis. Exclusion criteria encompassed patients with: (a) a history of any other cancer prior to HNSCC diagnosis, (b) distant metastasis, (c) previous treatment, including neoadjuvant chemotherapy, radiotherapy, or concurrent chemoradiotherapy prior to radical surgery, (d) autoimmune diseases, hematological disorders, or anticoagulation therapy, (e) clinical evidence of acute inflammatory liver diseases, acute biliary tract infection, or acute pancreatitis within 4 weeks prior to blood tests, and (f) unavailable pretreatment serum albumin and total bilirubin values. This study was approved by the Medical Ethics Committee and Human Clinical Trial Committee, Chang Gung Memorial Hospital (Ethical Application Reference number: 202300640B0) while patients' consent to review their medical records was not required by this hospital's committees because the patient data remained anonymous.

### 2.2 Study Design and Data Collection

The 663 enrolled patients were randomly divided into training and validation cohorts in a 3:1 ratio. The predictive model was constructed based on patient characteristics and survival outcomes from the training cohort and subsequently validated using the validation cohort. Data on clinical features and pathological characteristics, including gender, age, lifestyle factors, primary cancer site, pathological stage, perineural invasion (PNI), lymphovascular invasion (LVI), histological grade, extranodal extension (ENE), surgical margin status, and type of adjuvant therapy, were collected and analyzed for survival impact. Pathological staging was reclassified according to the 8th edition of the AJCC system.

Preoperative ALBI scores were calculated using the formula: log10 (serum total bilirubin [μmol/L]) × 0.66 + serum albumin [g/L] × −0.085, based on data collected within 1 week before surgery. The optimal ALBI cut‐off value for survival prediction was determined using X‐tile software (version 3.6.1) [25], stratifying patients into low‐ and high‐ALBI groups. The primary outcome was 5‐year overall survival (OS), calculated from the date of surgery to death or last follow‐up.

### 2.3 Statistical Analysis

Statistical analyses were conducted using R Studio and SPSS version 25.0 (IBM Corp.). Survival outcomes were estimated via the Kaplan–Meier method, with the log‐rank test used to determine significance. Associations between predictors and survival were assessed through univariate and multivariate Cox regression analyses, reporting hazard ratios (HRs) and 95% confidence intervals (CIs). A two‐tailed *p* < 0.05 indicated statistical significance.

Based on independent prognostic factors from multivariate analysis, a predictive nomogram was developed in the training cohort using the “rms” package (Version 5.1–0, Vanderbilt University, Nashville, TN, USA) in R. Calibration plots and the concordance index (C‐index) were used to assess model performance by comparing predicted and observed 5‐year OS. Internal validation was conducted via bootstrap resampling, and the discriminative ability of the model was further assessed using time‐dependent receiver operating characteristic (tROC) curves in both the training and validation cohorts.

## 3 Results

This study enrolled 663 patients, who were randomly divided into a training cohort (*n* = 497) and a validation cohort (*n* = 166) at a 3:1 ratio. As shown in Table 1, the baseline clinicopathological characteristics were well‐balanced between the two groups. Most patients were male, comprising 93.2% of the training cohort and 94.6% of the validation cohort. The most common tumor subsite in the training cohort was the oral cavity (*n* = 364, 73.2%), followed by the larynx (*n* = 51, 10.3%), oropharynx (*n* = 47, 9.5%), and hypopharynx (*n* = 35, 7%). In the validation cohort, the most common tumor subsite was the oral cavity (*n* = 120, 72.3%), followed by the oropharynx (*n* = 26, 15.7%), hypopharynx (*n* = 12, 7.2%), and larynx (*n* = 8, 4.8%). In the training cohort, there were 241 patients with early‐stage disease and 256 patients with advanced‐stage disease, while in the validation cohort, 83 patients had early‐stage disease and 83 patients had advanced‐stage disease. The presence of PNI was found in 146 patients (29.4%) in the training cohort and in 47 patients (28.3%) in the validation cohort. LVI was observed in 88 patients (17.7%) in the training cohort and 25 patients (15.1%) in the validation cohort. ENE was present in 79 patients (15.9%) in the training cohort and 24 patients (14.5%) in the validation cohort. Additionally, 229 patients (46.1%) in the training cohort and 85 patients (51.2%) in the validation cohort received adjuvant therapy postoperatively.

**TABLE 1:** Patient characteristics.

Characteristics	Training cohort (*N* = 497)	Validation cohort (*N* = 166)	*p*
	Value	Value	
Median Age (range), year	54 [31, 95]	53 [30, 86]	0.273
Median follow up time (range), months	68.1 [0.4, 140.8]	70.3 [4.2, 142.5]	0.216
Median value of preoperative ALBI (range)	−3.07 [−3.76, −1.62]	−3.07 [−3.89, −1.68]	0.819
Sex			
Male	463 (93.2%)	157 (94.6%)	0.52
Female	34 (6.8%)	9 (5.4%)	
Smoking habit			
Yes	439 (88.3%)	145 (87.3%)	0.736
Betel nut chewing			
Yes	396 (79.7%)	140 (84.3%)	0.187
Alcohol drinking			
Yes	396 (79.7%)	128 (77.1%)	0.481
Tumor location			
Oral cavity	364 (73.2%)	120 (72.3%)	0.811
Other subsites	133 (26.8%)	46 (27.7%)	
Pathological stage			
I–II	241 (48.5%)	83 (50%)	0.736
III–IV	256 (51.5%)	83 (50%)	
Histologic grade			
WDSCC	187 (37.6%)	67 (40.4%)	0.413
MDSCC	291 (58.6%)	96 (57.8%)	
PDSCC	19 (3.8%)	3 (1.8%)	
Perineural invasion			
Absent	351 (70.6%)	119 (71.7%)	0.794
Present	146 (29.4%)	47 (28.3%)	
Lymphovascular invasion			
Absent	409 (82.3%)	141 (84.9%)	0.432
Present	88 (17.7%)	25 (15.1%)	
Surgical margin			
< 5 mm	211 (42.5%)	63 (38.0%)	0.308
≧ 5 mm	286 (57.5%)	103 (62.0%)	
Extranodal extension			
Absent	418 (84.1%)	142 (85.5%)	0.658
Present	79 (15.9%)	24 (14.5%)	
Preoperative ALBI			
Low	383 (77.1%)	129 (77.7%)	0.863
High	114 (22.9%)	37 (22.3%)	
Treatment modality			
Surgery	268 (53.9%)	81 (48.8%)	0.462
Surgery then RT	85 (17.1%)	34 (20.5%)	
Surgery then CRT	144 (29.0%)	51 (30.7%)	

Abbreviations: ALBI: albumin‐bilirubin score; CRT: chemoradiotherapy; MDSCC: moderately differentiated squamous cell carcinoma; PDSCC: poorly differentiated squamous cell carcinoma; RT: radiotherapy; WDSCC: well differentiated squamous cell carcinoma.

The median values of preoperative ALBI score in the training cohort and validation cohort were −3.07 (range: −3.76 ~ −1.62) and −3.07 (range: −3.89 ~ −1.68), respectively. When ALBI was analyzed as a continuous variable, statistically significant trends were observed in both univariate and multivariate analyses. Elevated preoperative ALBI scores were associated with increased overall mortality in both the training cohort (adjusted HR: 2.276, 95% CI: 1.303–3.978, *p* = 0.004) and the validation cohort (adjusted HR: 3.090, 95% CI: 1.167–8.178, *p* = 0.023) (Tables S1 and S2).

**FIGURE 1:**
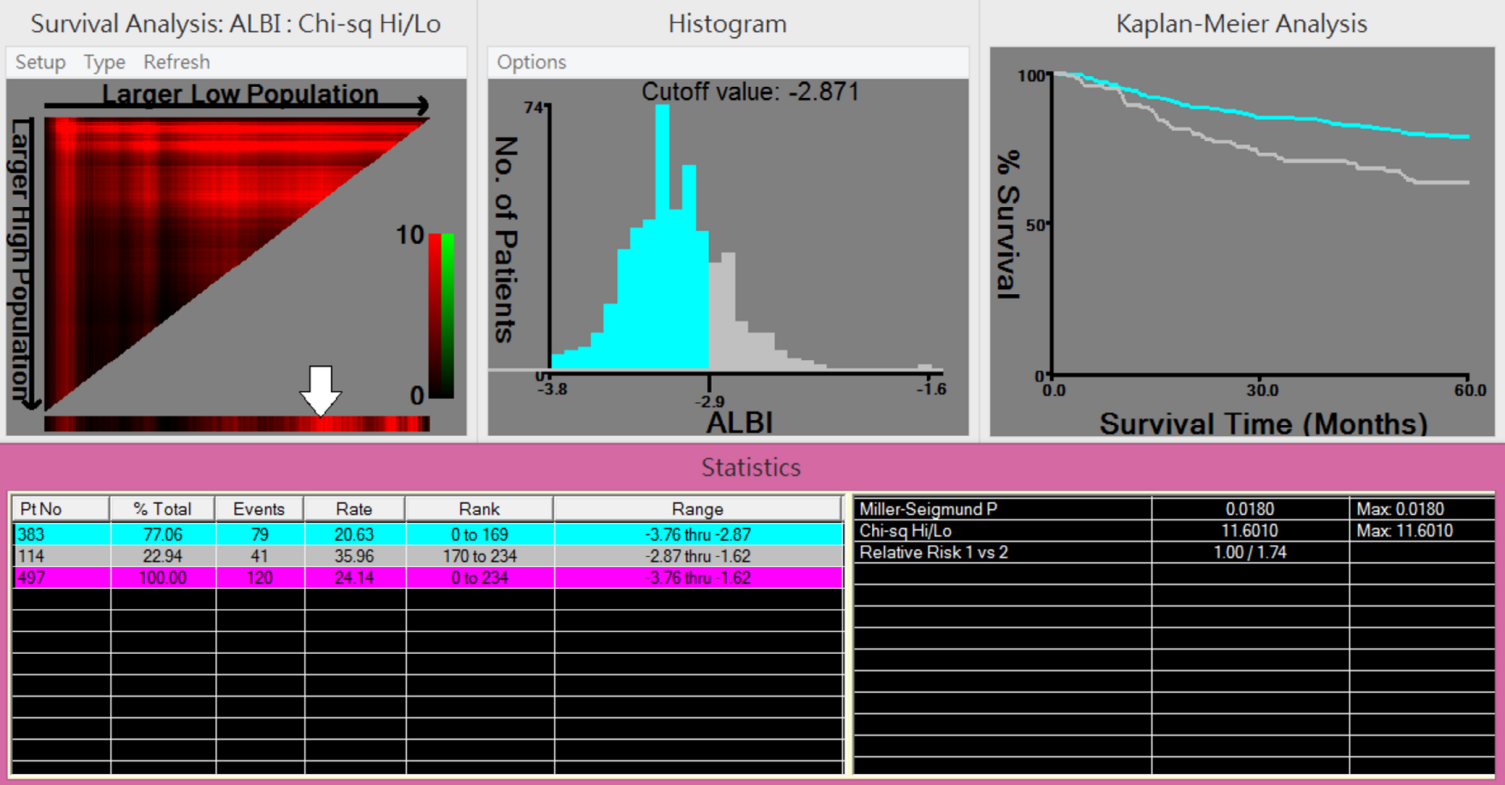
X‐tile analysis identifying the optimal preoperative ALBI cutoff based on overall survival (OS) in the training cohort. Upper left panels: X‐tile plots showing red coloration for inverse correlation with survival. The optimal cut‐point, corresponding to the brightest pixel, is indicated by a white arrow and represents the threshold yielding the most significant survival difference (*χ* ^2^ = 11.6010, *p* = 0.0180). The optimal cutoff value was determined to be −2.871. Middle panel: Histogram of ALBI scores. Right panel: Kaplan–Meier survival curves based on the identified cutoff. Because the histogram *X*‐axis shows only one decimal place, the precise threshold is annotated as “Cutoff value = –2.871.”

We subsequently applied the established ALBI grading system [17] to categorize patients. However, in the training cohort, 464 patients (93.4%) were classified as Grade 1 and only 33 (6.6%) as Grade 2, with no patients falling into Grade 3. A similar distribution was observed in the validation cohort, with 156 patients (94.0%) in Grade 1 and 10 (6.0%) in Grade 2, again with no Grade 3 cases. This pronounced skew toward Grade 1 substantially limited the statistical power and discriminative capacity of the standard grading system in our population. To better stratify prognostic risk within our cohort, we therefore employed X‐tile software to derive an optimal cut‐off value. X‐tile analysis identified −2.871 as the optimal ALBI score cutoff for predicting 5‐year OS in the training cohort (Figure 1).

Patients were subsequently divided into high (ALBI ≥ −2.871) and low (ALBI < −2.871) ALBI groups in both the training (*n* = 114 and *n* = 383, respectively) and validation cohorts (*n* = 37 and *n* = 129, respectively). In the training cohort, a high ALBI score was significantly correlated with older age and higher histological grade, while in the validation cohort, it was associated with older age. These associations are detailed in Table 2.

**TABLE 2:** Associations between ALBI and multiple clinicopathological parameters.

Variable	Training cohort			Validation cohort		
	ALBI		*p*	ALBI		*p*
	Low	High		Low	High	
Age						
> 60	95	47	**0.001**	30	16	**0.017**
≤ 60	288	67		99	21	
Sex						
Male	355	108	0.447	122	35	1.000
Female	28	6		7	2	
Smoking						
No	46	12	0.665	14	7	0.259
Yes	337	102		115	30	
Betel nut chewing						
No	81	20	0.401	18	8	0.258
Yes	302	94		111	29	
Alcohol drinking						
No	72	29	0.122	26	12	0.117
Yes	311	85		103	25	
Cancer location						
Oral cavity	285	79	0.279	93	27	0.916
Other subsites	98	35		36	10	
Pathological stage						
I + II	194	47	0.077	64	19	0.852
III + IV	189	67		65	18	
Histologic grade						
WDSCC	133	54	**0.014**	52	15	0.980
MDSCC/PDSCC	250	60		77	22	
Perineural invasion						
Absent	265	86	0.199	92	27	0.844
Present	118	28		37	10	
Lymphovascular invasion						
Absent	317	92	0.612	111	30	0.457
Present	66	22		18	7	
Extranodal extension						
Absent	321	97	0.744	111	31	0.73
Present	62	17		18	6	
Surgical margin						
< 5 mm	164	47	0.763	52	11	0.242
≧ 5 mm	219	67		77	26	
Treatment modality						
Surgery	208	60	0.416	65	16	0.708
Surgery then RT	61	24		25	9	
Surgery then CRT	114	30		39	12	

*Note*: Numbers in bold Indicate statistically significant values.

Abbreviations: ALBI: albumin‐bilirubin score; CRT: chemoradiotherapy; MDSCC: moderately differentiated squamous cell carcinoma; PDSCC: poorly differentiated squamous cell carcinoma; RT: radiotherapy; WDSCC: well‐differentiated squamous cell carcinoma.

### 3.1 Prognostic Value of ALBI in HNSCC Patients

The overall median follow‐up duration was 68.6 months, ranging from 0.4 to 142.5 months. In the training cohort, treatment failure occurred in 104 patients (20.9%), while 30 patients (18.1%) experienced treatment failure in the validation cohort. A total of 192 all‐cause death events were recorded, comprising 144 events in the training cohort and 48 in the validation cohort. The 5‐year OS rates were 75.4% in the training cohort and 80.6% in the validation cohort.

**FIGURE 2:**
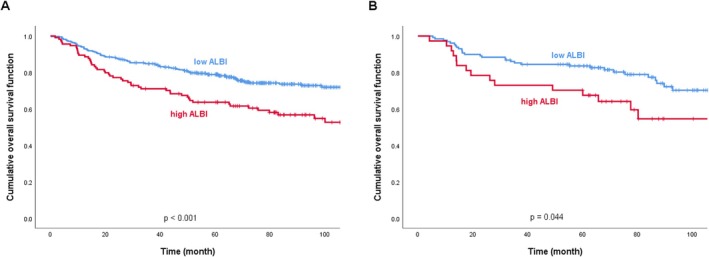
Kaplan–Meier survival curves. Kaplan–Meier survival curves according to different preoperative ALBI values. (a) Training cohort, and (b) validation cohort.

Kaplan–Meier survival curves demonstrated that patients in the high‐ALBI group had significantly worse outcomes compared to those in the low‐ALBI group, in both training and validation cohorts. The 5‐year OS rate in the training cohort was significantly lower in the high‐ALBI group (63.6%) than in the low‐ALBI group (78.9%; *p* < 0.001), while a similar trend was observed in the validation cohort, with 5‐year OS rates of 70.3% and 83.6% for the high‐ and low‐ALBI groups, respectively (*p* = 0.044) (Figure 2).

### 3.2 Independent Prognosticators of OS

In the training cohort, preoperative ALBI score, age, cancer location, pathological stage, LVI, PNI, histological grade, surgical margin status, ENE, and need for adjuvant therapy were significant in univariate Cox regression (*p* < 0.05). High ALBI was still found to be an independent negative prognosticator of OS (HR: 1.776, 95% CI: [1.24–2.544], *p =* 0.002) compared to low ALBI in multivariate Cox regression analysis. Advanced pathological stage, older age, inadequate surgical margin, aggressive histological grade, and presence of ENE also remained significant adverse prognosticators (all *p* < 0.05) (Table 3). Similarly, multivariate analysis in the validation cohort confirmed that high ALBI score and presence of ENE were independent negative prognosticators of OS (both *p* < 0.05) (Table 4).

**TABLE 3:** Univariate and multivariate analysis of factors impacting survival in the training cohort.

Variables	Event	5‐year OS (%)	Univariate Cox analysis		Multivariate Cox analysis	
			Hazard ratio (95% CI)	*p*	Hazard ratio (95% CI)	*p*
Age						
≤ 60	91	78.7	1	**0.004**	1	**0.032**
> 60	53	67.0	1.654 (1.178, 2.323)		1.473 (1.035, 2.097)	
Sex						
Male	133	75.6	1	0.519	N/A	
Female	11	72.8	1.224 (0.662, 2.265)			
Smoking						
No	17	75.4	1	0.856	N/A	
Yes	127	75.4	0.954 (0.575, 1.583)			
Betel nut chewing						
No	30	72.9	1	0.742	N/A	
Yes	114	76.0	0.935 (0.625, 1.398)			
Alcohol drinking						
No	33	70.0	1	0.23	N/A	
Yes	111	76.7	0.788 (0.534, 1.163)			
Pathological stage						
I + II	31	90.0	1	**< 0.001**	1	**< 0.001**
III + IV	113	61.6	4.33 (2.909, 6.447)		3.348 (2.003, 5.598)	
Cancer location						
Oral cavity	90	78.5	1	**0.001**	1	0.971
Other subsites	54	66.4	1.779 (1.269, 2.494)		1.007 (0.687, 1.475)	
Histologic grade						
WDSCC	33	85.2	1	**< 0.001**	1	**0.009**
MDSCC/PDSCC	111	69.4	2.406 (1.63, 3.553)		1.758 (1.149, 2.688)	
Perineural invasion						
Absent	84	80.4	1	**< 0.001**	1	0.888
Present	60	63.1	1.954 (1.403, 2.722)		1.028 (0.703, 1.503)	
Lymphovascular invasion						
Absent	98	80.0	1	**< 0.001**	1	0.144
Present	46	53.8	2.837 (1.995, 4.034)		1.351 (0.902, 2.023)	
Extranodal extension						
Absent	101	79.9	1	**< 0.001**	1	**0.048**
Present	43	51.4	3.114 (2.177, 4.454)		1.518 (1.003, 2.299)	
Surgical margin						
≧5 mm	70	78.3	1	**0.011**	1	**0.039**
< 5 mm	74	71.3	1.529 (1.102, 2.12)		1.437 (1.019, 2.028)	
Preoperative ALBI						
Low	94	78.9	1	**< 0.001**	1	**0.002**
High	50	63.6	1.903 (1.35, 2.682)		1.776 (1.24, 2.544)	
Treatment modality						
Surgery	45	86.2	1	**< 0.001**	1	0.952
Surgery then RT	34	66.8	2.473 (1.584, 3.861)		0.92 (0.543, 1.557)	
Surgery then CRT	65	60.5	3.363 (2.298, 4.922)		0.939 (0.554, 1.593)	

*Note*: Numbers in bold Indicate statistically significant values.

Abbreviations: ALBI: albumin‐bilirubin score; CRT: chemoradiotherapy; MDSCC: moderately differentiated squamous cell carcinoma; N/A: not applicable; OS: overall survival; PDSCC: poorly differentiated squamous cell carcinoma; RT: radiotherapy; WDSCC: well‐differentiated squamous cell carcinoma.

**TABLE 4:** Univariate and multivariate analysis of factors impacting survival in validation cohort.

Variable	Event	5‐year OS (%)	Univariate Cox analysis		Multivariate Cox analysis	
			Hazard ratio (95% CI)	*p*	Hazard ratio (95% CI)	*p*
Age						
≤ 60	34	81.5	1	0.427	N/A	
> 60	14	78.3	0.775 (0.413, 1.454)			
Sex						
Male	46	80.8	1	0.671	N/A	
Female	2	77.8	0.735 (0.178, 3.04)			
Smoking						
No	4	85.7	1	0.353	N/A	
Yes	44	79.9	1.625 (0.583, 4.528)			
Betel nut chewing						
No	6	88.5	1	0.427	N/A	
Yes	42	79.2	1.416 (0.6, 3.338)			
Alcohol drinking						
No	14	73.7	1	0.392	N/A	
Yes	34	82.7	0.756 (0.398, 1.434)			
Pathological stage						
I + II	14	92.8	1	**0.001**	1	0.3
III + IV	34	68.6	3.11 (1.641, 5.896)		1.631 (0.646, 4.119)	
Cancer location						
Oral cavity	30	83.3	1	0.09	N/A	
Other subsites	18	73.4	1.665 (0.924, 3)			
Histologic grade						
WDSCC	19	80.5	1	0.501	N/A	
MDSCC/PDSCC	29	80.8	1.225 (0.679, 2.209)			
Perineural invasion						
Absent	29	84.7	1	**0.01**	1	0.608
Present	19	70.2	2.152 (1.199, 3.865)		1.21 (0.585, 2.502)	
Lymphovascular invasion						
Absent	36	83.6	1	**0.005**	1	0.43
Present	12	64.0	2.585 (1.337, 4.996)		1.352 (0.639, 2.86)	
Extranodal extension						
Absent	34	86.5	1	**< 0.001**	1	**0.041**
Present	14	45.8	3.87 (2.064, 7.255)		2.313 (1.037, 5.16)	
Surgical margin						
≧ 5 mm	28	82.4	1	0.648	N/A	
< 5 mm	20	77.8	1.145 (0.639, 2.052)			
Preoperative ALBI						
Low	32	83.6	1	**0.047**	1	**0.038**
High	16	70.3	1.862 (1.007, 3.443)		1.965 (1.038, 3.722)	
Treatment modality						
Surgery	12	95.1	1	**< 0.001**	1	0.322
Surgery then RT	12	73.3	2.846 (1.254, 6.46)		2.052 (0.792, 5.314)	
Surgery then CRT	24	62.7	4.291 (2.1, 8.767)		1.592 (0.475, 5.34)	

*Note*: Numbers in bold indicate statistically significant values.

Abbreviations: ALBI: albumin‐bilirubin score; CRT: chemoradiotherapy; MDSCC: moderately differentiated squamous cell carcinoma; N/A: not applicable; OS: overall survival; PDSCC: poorly differentiated squamous cell carcinoma; RT: radiotherapy; WDSCC: well‐differentiated squamous cell carcinoma.

### 3.3 Development of a Novel ALBI‐Based Prognostic Model

**FIGURE 3:**
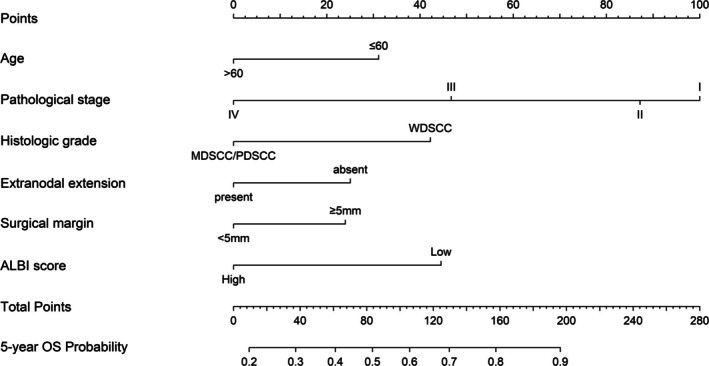
Nomogram and survival predictions. Nomogram for predicted overall survival (OS) rate. A vertical line is drawn from each factor to the point score. By adding the points from all the factors, a total points score is reached, which is translated into 5‐year OS rates by drawing a vertical line to its axis.

**FIGURE 4:**
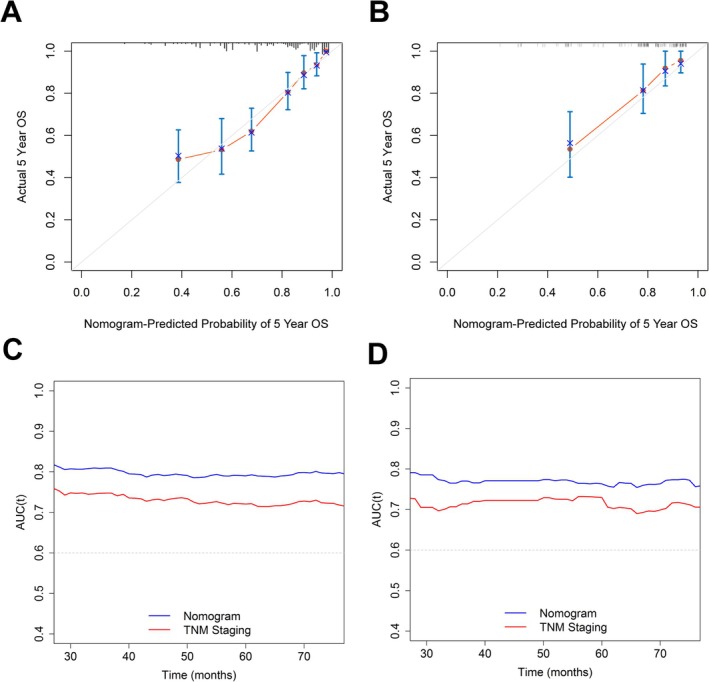
The calibration and comparison of the predictive capabilities of this ALBI‐based prognostic model. (a) Calibration plot in the training cohort. (b) Calibration plot in the validation cohort. (c) Time‐dependent ROC curves comparison between the current prognostic model and the traditional TNM staging system in the training cohort. (d) Time‐dependent ROC curves comparison between the current prognostic model and the traditional TNM staging system in the validation cohort.

A novel prognostic model was constructed by incorporating the significant prognostic factors identified in the training cohort to enhance survival prediction accuracy. Among these factors, pathological stage contributed the most to prognosis, followed by ALBI score, histological grade, age, ENE, and surgical margin status (Figure 3). Each prognostic factor in the nomogram was assigned a point value, and the total points were summed to calculate an individual patient's score. The 5‐year OS probability was subsequently determined by referencing the survival probability scale based on the total score. Calibration plots (Figure 4A,B) demonstrated strong concordance between predicted and observed 5‐year OS in both the training and validation cohorts, confirming high accuracy of the proposed model. The nomogram had a C‐index of 0.759 (95% CI: 0.724–0.794) in the training cohort and 0.749 (95% CI: 0.671–0.827) in the validation cohort, significantly outperforming the traditional TNM system (C‐index: 0.703 and 0.684, respectively; training cohort: *p* < 0.001; validation cohort: *p* = 0.001). Time‐dependent ROC curve analysis (Figure 4C,D) further confirmed that the ALBI‐based model outperformed the traditional TNM staging system, highlighting its utility for clinical practice and risk stratification in HNSCC patients.

## 4 Discussion

To our knowledge, this is the first study to evaluate the prognostic value of the ALBI score in HNSCC patients, offering new perspectives on its application in disease management. First, we observed a significant association between higher ALBI scores and poorer OS rates in both training and validation cohorts. Second, upon stratifying patients into low‐ALBI (< −2.871) and high‐ALBI (≥ −2.871) groups, multivariate analysis identified a high ALBI score as an independent negative prognosticator for outcomes in both cohorts. Thirdly, the prognostic nomogram incorporating the ALBI score demonstrated superior performance compared to the traditional TNM staging system, suggesting that this ALBI‐based model could serve as a convenient, non‐invasive, affordable, and reliable tool to enhance prognostic prediction and assist clinicians in treatment decision‐making for HNSCC patients undergoing curative surgery.

The ALBI score, combining both serum bilirubin and albumin levels, was originally developed as an index for evaluating reserved liver function in patients with HCC. It could be divided into three grades according to nutrition‐related risk conventionally (Grade 1: ≤ −2.60; Grade 2: more than −2.60 to ≤ −1.39; Grade 3: > −1.39). The prognostic role of ALBI grade in various human malignancies has been reported in recent years. Imamura et al. demonstrated that patients with pretreatment ALBI grade 1 had better OS and relapse‐free survival than two other groups in pancreatic adenocarcinoma [20]. Kanda et al. studied 283 patients with gastric cancer and reported that the preoperative ALBI Grade 2 group was more likely to have shorter cancer‐specific and disease‐free survival compared with the ALBI Grade 1 group [21]. Another study also showed pretreatment ALBI grade was one of the independent predictors of progression‐free survival and OS in patients with advanced or recurrent non‐small‐cell lung cancer treated with cancer immunotherapy [24]. Some studies have analyzed the importance of ALBI score in different types of cancer by continuous values of ALBI score; to wit: Lee et al. analyzed 510 patients with stage III colon cancer and also revealed those with preoperative ALBI score less than −2.54 were significantly associated with better cancer‐specific and disease‐free survival than those with an ALBI > −2.54 [26]; besides, Aoyama et al. analyzed the association between ALBI score and prognosis in 121 patients with esophageal cancer and found that patients with a higher ALBI score (ALBI > −2.7) had poorer OS and recurrence‐free survival than those with a lower ALBI score [23]; furthermore, Zhang et al. found the optimal cut‐off value of ALBI to be −2.941 for prediction of OS and progression‐free survival in 194 patients diagnosed with high‐grade gliomas [27].

In head and neck cancers, Wang et al. retrospectively analyzed 259 patients with HNSCC who received curative treatment including surgery, definitive radiotherapy, and concurrent chemoradiotherapy [28], demonstrating that the pretreatment ALBI score had significant prognostic value for both OS and recurrence‐free survival (RFS) in both training and validation cohorts. In contrast, our study focused on a more homogeneous cohort of HNSCC patients who all underwent curative surgery as the initial and primary treatment, without prior radiotherapy or chemoradiotherapy. To further expand our findings, we evaluated the prognostic value of the preoperative ALBI score for disease‐free survival (DFS), defined as the time from surgery to recurrence, death from any cause, or last follow‐up. In the study cohort, a significant association was found between the continuous ALBI score and DFS (HR = 2.259, 95% CI: 1.517–3.362; *p* < 0.001), where using −2.871 as the cutoff, patients with high ALBI scores were found to have significantly lower 5‐year DFS compared to those with low ALBI scores (60% vs. 72.5%, *p* < 0.001).

A high ALBI score likely reflects a combination of relative hyperbilirubinemia and hypoalbuminemia. Both serum albumin and bilirubin are crucial indicators of liver function and are routinely assessed at various time points, including diagnosis, pre‐treatment evaluation, and during follow‐up. Albumin, synthesized by the liver, serves not only as a marker of liver function but also as an indicator of nutritional status. Hypoalbuminemia may reflect not only hepatic dysfunction but also increased protein consumption due to systemic disease processes, particularly those associated with aggressive tumor proliferation [29]. Meanwhile, hypoalbuminemia may impair the metabolism and the function of immune cells, subsequently resulting in a weakened immune reaction, occurrence of infectious disease, and poor response to anti‐cancer treatment [30]. Several studies have shown that pretreatment hypoalbuminemia is associated with poorer oncologic outcomes in patients with HNSCC [31, 32]; on the other hand, serum bilirubin has not yet been clearly associated with the prognosis of HNSCC. Serum bilirubin, regarded as the end product of heme metabolism, had been considered to have no physiological function previously, although recent data suggested endogenous bilirubin could be one of the powerful signaling molecules and reserved as an activator of aryl hydrocarbon receptor (AhR) [33, 34], which plays an important role in modulating different inflammatory blood cells, including regulatory T cells (Treg), T‐helper 17 cells (Th17), and B cells [35, 36]. Besides, AhR could direct macrophage polarization in cancer cells and suppress inflammatory T cell infiltration while resulting in tumor growth and worse outcomes [37]. These studies can accordingly help explain the prognostic role of ALBI score for patients with cancer.

Additionally, our findings revealed that ENE was also determined to be another independent risk factor for OS in both training and validation cohorts. ENE is known as a pathological phenomenon in which extension of metastatic tumor occurs through the lymph node capsule into the surrounding connective tissue and is also a common risk factor for locoregional recurrences and poor survival chances in patients with HNSCC [38]. Our previous studies also demonstrated that the presence of ENE in the ipsilateral neck had significantly increased the risk of occult contralateral neck metastasis in patients with p16‐negative oropharyngeal cancer who had been treated with surgery using bilateral neck dissection [39].

However, several limitations of our study should be acknowledged. First, this is a retrospective, single‐institute study and all patients underwent surgical procedures performed by different head and neck surgeons. Second, our cohort focused on patients' upfront surgery with/without adjuvant therapy, so our results might fail to extend to the patient's upfront definitive radiotherapy or chemoradiotherapy; accordingly, the study is prone to experience selection bias. Third, the study's findings were not validated using an external dataset. Future studies, designed prospectively and conducted across multiple institutions, might help to minimize these biases and provide more robust validation.

In summary, the study underscores the promise of the preoperative ALBI score as a prognostic biomarker for individuals with HNSCC. Given the ease of obtaining the ALBI score from routine preoperative laboratory tests, we advocate its potential integration into routine clinical practice and oncological research.

## Conflicts of Interest

The authors declare no conflicts of interest.

## Supporting information


**Table S1:** Univariate and multivariate analysis of factors impacting overall survival in training cohort while ALBI in continuous data.
**Table S2:** Univariate and multivariate analysis of factors impacting overall survival in validation cohort while ALBI in continuous data.

## Data Availability

The data that support the findings of this study are available from the corresponding author upon reasonable request.
