# Supplementary material for: Albumin‐Bilirubin Score as a Novel Prognostic Prediction Tool for Surgically Treated Head and Neck Squamous Cell Carcinoma
Source: Kaohsiung J Med Sci. 2025 Sep 25;42(4):e70115. doi: 10.1002/kjm2.70115 (PMC13147938; doi:10.1002/kjm2.70115)
Supplement: Supplementary file 1 — Table S1: Univariate and multivariate analysis of factors impacting overall survival in training cohort while ALBI in continuous data. Table S2: Univariate and multivariate analysis of factors impacting overall survival in validation cohort while ALBI in continuous data. [file KJM2-42-e70115-s001.docx]

| Table S1. Univariate and multivariate analysis of factors impacting overall survival in training cohort while ALBI in continuous data. | | | | | | |
| --- | --- | --- | --- | --- | --- | --- |
| Variable | | Event | Univariate Cox analysis | | Multivariate Cox analysis | |
|  |  |  | Hazard ratio (95% CI) | *p*-value | Hazard ratio (95% CI) | *p*-value |
| Age | ≤ 60 | 91 | 1 | **0.004** | 1 | **0.021** |
|  | > 60 | 53 | 1.654 (1.178, 2.323) |  | 1.508 (1.064, 2.138) |  |
| Sex | male | 133 | 1 | 0.519 | N/A | |
|  | female | 11 | 1.224 (0.662, 2.265) |  |  |  |
| Smoking | No | 17 | 1 | 0.856 | N/A | |
|  | Yes | 127 | 0.954 (0.575, 1.583) |  |  |  |
| betel nut chewing | No | 30 | 1 | 0.742 | N/A | |
|  | Yes | 114 | 0.935 (0.625, 1.398) |  |  |  |
| Alcohol drinking | No | 33 | 1 | 0.23 | N/A | |
|  | Yes | 111 | 0.788 (0.534, 1.163) |  |  |  |
| Pathological stage | I+II | 31 | 1 | **< 0.001** | 1 | **< 0.001** |
|  | III+IV | 113 | 4.33 (2.909, 6.447) |  | 3.362 (2.007, 5.633) |  |
| Cancer location | oral cavity | 90 | 1 | **0.001** | 1 | 0.907 |
|  | other subsites | 54 | 1.779 (1.269, 2.494) |  | 0.977 (0.665, 1.437) |  |
| Histologic grade | WDSCC | 33 | 1 | **< 0.001** | 1 | **0.01** |
|  | MDSCC/PDSCC | 111 | 2.406 (1.63, 3.553) |  | 1.746 (1.141, 2.67) |  |
| Perineural invasion | absent | 84 | 1 | **< 0.001** | 1 | 0.841 |
|  | present | 60 | 1.954 (1.403, 2.722) |  | 1.04 (0.71, 1.523) |  |
| Lymphovascular invasion | absent | 98 | 1 | **< 0.001** | 1 | 0.161 |
|  | present | 46 | 2.837 (1.995, 4.034) |  | 1.339 (0.89, 2.016) |  |
| Extranodal extension | absent | 101 | 1 | **< 0.001** | 1 | 0.051 |
|  | present | 43 | 3.114 (2.177, 4.454) |  | 1.515 (0.998, 2.301) |  |
| Surgical margin | ≧5mm | 70 | 1 | **0.011** | 1 | 0.056 |
|  | <5mm | 74 | 1.529 (1.102, 2.12) |  | 1.398 (0.992, 1.971) |  |
| Preoperative ALBI (in continuous data) | | 144 | 2.647 (1.64, 4.27) | **< 0.001** | 2.276 (1.303, 3.978) | **0.004** |
| Treatment modality | surgery | 45 | 1 | **< 0.001** | 1 |  |
|  | surgery then RT | 34 | 2.473 (1.584, 3.861) |  | 0.843 (0.494, 1.437) | 0.816 |
|  | surgery then CRT | 65 | 3.363 (2.298, 4.922) |  | 0.917 (0.538, 1.562) |  |
| Number in bold indicates statistically significant values. | | | | | | |
| Abbreviation: WDSCC: well-differentiated squamous cell carcinoma; MDSCC: moderately-differentiated squamous cell carcinoma; PDSCC: poorly-differentiated squamous cell carcinoma; ALBI: albumin-bilirubin score; RT: radiotherapy; CRT: chemoradiotherapy; N/A: not applicable. | | | | | | |

| Table S2. Univariate and multivariate analysis of factors impacting overall survival in validation cohort while ALBI in continuous data. | | | | | | |
| --- | --- | --- | --- | --- | --- | --- |
| Variable | | Event | Univariate Cox analysis | | Multivariate Cox analysis | |
|  |  |  | Hazard ratio (95% CI) | *p*-value | Hazard ratio (95% CI) | *p*-value |
| Age | ≤ 60 | 34 | 1 | 0.427 | 1 | 0.241 |
|  | > 60 | 14 | 0.775 (0.413, 1.454) |  | 1.5 (0.761, 2.955) |  |
| Sex | male | 46 | 1 | 0.671 | N/A | |
|  | female | 2 | 0.735 (0.178, 3.04) |  |  |  |
| Smoking | No | 4 | 1 | 0.353 | N/A | |
|  | Yes | 44 | 1.625 (0.583, 4.528) |  |  |  |
| betel nut chewing | No | 6 | 1 | 0.427 | N/A | |
|  | Yes | 42 | 1.416 (0.6, 3.338) |  |  |  |
| Alcohol drinking | No | 14 | 1 | 0.392 | N/A | |
|  | Yes | 34 | 0.756 (0.398, 1.434) |  |  |  |
| Pathological stage | I+II | 14 | 1 | **0.001** | 1 | 0.369 |
|  | III+IV | 34 | 3.11 (1.641, 5.896) |  | 1.532 (0.604, 3.887) |  |
| Cancer location | oral cavity | 30 | 1 | 0.09 | 1 | 0.535 |
|  | other subsites | 18 | 1.665 (0.924, 3) |  | 1.257 (0.61, 2.593) |  |
| Histologic grade | WDSCC | 19 | 1 | 0.501 | 1 | 0.653 |
|  | MDSCC/PDSCC | 29 | 1.225 (0.679, 2.209) |  | 0.854 (0.429, 1.7) |  |
| Perineural invasion | absent | 29 | 1 | **0.01** | 1 | 0.523 |
|  | present | 19 | 2.152 (1.199, 3.865) |  | 1.282 (0.598, 2.748) |  |
| Lymphovascular invasion | absent | 36 | 1 | **0.005** | 1 | 0.245 |
|  | present | 12 | 2.585 (1.337, 4.996) |  | 1.644 (0.711, 3.8) |  |
| Extranodal extension | absent | 34 | 1 | **< 0.001** | 1 | **0.035** |
|  | present | 14 | 3.87 (2.064, 7.255) |  | 2.422 (1.066, 5.504) |  |
| Surgical margin | ≧5mm | 28 | 1 | 0.648 | 1 | 0.453 |
|  | <5mm | 20 | 1.145 (0.639, 2.052) |  | 0.772 (0.394, 1.516) |  |
| Preoperative ALBI (in continuous data) | | 48 | 3.131 (1.206, 8.131) | **0.019** | 3.09 (1.167, 8.178) | **0.023** |
| Treatment modality | surgery | 12 | 1 | **< 0.001** | 1 |  |
|  | surgery then RT | 12 | 2.846 (1.254, 6.46) |  | 2.183 (0.816, 5.837) | 0.292 |
|  | surgery then CRT | 24 | 4.291 (2.1, 8.767) |  | 1.687 (0.514, 5.534) |  |
| Number in bold indicates statistically significant values. | | | | | | |
| Abbreviation: WDSCC: well-differentiated squamous cell carcinoma; MDSCC: moderately-differentiated squamous cell carcinoma; PDSCC: poorly-differentiated squamous cell carcinoma; ALBI: albumin-bilirubin score; RT: radiotherapy; CRT: chemoradiotherapy; N/A: not applicable. | | | | | | |
